# Case report: Vulvar metastasis after lung metastasis in cervical squamous cell carcinoma, a case report and literature review

**DOI:** 10.1016/j.ijscr.2025.110816

**Published:** 2025-01-04

**Authors:** Ze-lan Liao, Ka-na Wang, Jia-wen Zhang

**Affiliations:** a Department of Obstetrics and Gynecology, Clinical Medical College & Affiliated Hospital of Chengdu University, Chengdu, China; b Department of Obstetrics and Gynecology, West China Second University Hospital of Sichuan University, Chengdu, China; c Key Laboratory of Birth Defects and Related Diseases of Women and Children (Sichuan University), Ministry of Education, Chengdu, China

**Keywords:** Cervical squamous cell carcinoma, vulvar metastasis, HPV, false negative, therapy

## Abstract

**Introduction and importance:**

Cervical cancer is highly correlated with high-risk human papillomavirus (HPV) infection, accounting for approximately 70 % of cases. However, false-negative HPV test results can occur, complicating early detection.

**Case presentation:**

We introduce a rare case of cervical cancer with lung metastasis followed by vulvar metastasis. Notably, the HPV test was negative at diagnosis but became positive upon detection of vulvar metastasis.

**Clinical discussion:**

We discuss the pathways of vulvar metastasis in cervical cancer and potential causes of false-negative HPV tests. A search for cases of vulvar metastasis of cervical cancer in Medline and Embase between 2004 and 2024 was performed and a literature review was conducted.

**Conclusion:**

The occurrence of vulvar metastasis indicates the terminal manifestation of the tumor and the survival period is short. Active radiotherapy, chemotherapy, immunotherapy and other multiple treatments may increase the overall survival time of patients.

## Highlights

• Patients with cervical cancer were found to develop lung metastases and vulvar metastases.
• Cervical cancer found to be linked to high-risk HPV infections.
• Immunotherapy found to be effective in PD-L1-positive cervical cancer patients.
• Finding HPV infection in patients with cervical cancer is a progressive process

## 1 Introduction

Cervical cancer ranks as the fourth most common malignancy among women globally [1]. At present, scholars agree that the long-term and repeated infection of high-risk HPV is a necessary condition for the occurrence of cervical cancer [2]. In particular, HPV 16, followed by HPV 18, contributed to 70 % of HPV associated with cervical cancer as well as head and neck cancer [3]. Here we show a case of cervical squamous cell carcinoma initially presenting with negative HPV DeoxyriboNucleic Acid (DNA) results. However, subsequent tests revealed HPV 16 and HPV 58 positivity as well as PD-L1 expression, along with occurrence of lung metastasis and vulvar metastasis. At 19 months postoperatively, the patient remains under follow-up, with the main symptom of left leg swelling. To strengthen the understanding of cervical cancer with vulvar metastasis and avoid misdiagnosis, the literature review was also carried out (Table 1). The work has been reported in line with the SCARE criteria and PROCESS criteria [4].

**Table 1:** Reported cases of vulval metastasis in cervical malignancy.

Patient no.	Age	First author	Number of patients	FIGO stage and types of cancer cervix	Treatment taken	Appearance of lesions after therapy	Sites	Clinical manifestation	Outcome (months)
1	60	Agrawal [6]	1	IV, AC	CR + RT	4 months	V/A	N	D (13 months)
2	35	Srivastava K [7]	1	IIA, SCC	SUR + CR + RT	3.5 years	V	N	D (6 months)
3	39	Deepika Deka [8]	1	IIA, SCC	SUR + CR + RT	6.5 years	V	E, U	Poor prognosis (UK)
4	47	Grabiec M [9]	1	IB, SCC	SUR + RT	12 weeks	V	N	D (4 months)
5	46	Won-Jeong Kim [10]	1	IB, SCC	SUR + CR + RT	2.5 years	V	P, VE	D (6 months)
6	37	Richmond N A [11]	1	IB, AC	SUR + CR + RT	15 months	V	P, ERO	D (11 months)
7	50	Smriti Naswa [12]	1	IIIB, SCC	PAL+RT	Cervical cancer is diagnosed with skin symptoms	V	E, P, U	Poor prognosis (UK)
8	41	Hüseyin Çağlayan Özcan [13]	1	IB, SCC	SUR + CR + RT	5 months	V	N	D (11 months)
9	41	Javier Burbano [14]	1	IIIB, AC	CR + RT	1 year	V, LE	P, PER, U	D (13 months)

CR:chemoradiation, RT: radiation therapy, SUR: surgery, PAL: palliative treatment, SCC: squamous cell carcinoma, AC: adenocarcinoma, V: vulva, A: abdominal wall, LE: lower extremity, N: nodule, P: plaque, PER: perkeratosis, U: ulceration, E: edema, VE: vesicaes, ERO: erosions, D: dead, AL: alive, UK: unknown.

## 2 Case presentation

This is a 51-year-old Chinese Han woman, sought treatment at a local hospital after experiencing abnormal vaginal bleeding for two months. CT and colposcopic biopsy confirmed cervical squamous cell carcinoma. She has not engaged in unclean sexual activity and has not had more than one sexual partner. She had sex for the first time at the age of 21 and had always used condoms for birth control.

The patient went to our hospital for further evaluation. There were tumor-like lesions on the anterior and posterior lips of the cervix, about 4 cm–5 cm in size, and contact bleeding. In gynecological examination, the results of uterine body, bilateral fallopian tubes, ovaries and parauterine tissue examination were negative. Preoperative chest CT showed no abnormality. Preoperative HPV DNA was negative. The patient underwent total abdominal hysterectomy, bilateral salpingectomy, oophorectomy, pelvic lymph node dissection, and abdominal aortic lymph node sampling. The surgical pathological analysis of FIGO 2018 for postoperative staging of cervical cancer was diagnosed as cervical squamous cell carcinoma stage I B [5]. The positive rate of P16+ and Ki 67 was about 70 %. Post-operative tissue gene detection showed positive for programmed death receptor-1 (PD-L1).

The patient continued to receive 4 cycles of chemotherapy containing cisplatin and 25 times of external pelvic irradiation plus 2 times of three-dimensional afterloading radiotherapy. Follow-up included clinical evaluation and SCC examination, chest, abdomen and pelvic CT every 3 months. More than 9 months after operation, HPV-DNA was still negative.

At the beginning of 10 months after operation, SCC increased by routine examination. 12 months after operation, chest CT found multiple small nodules scattered in both lungs, and diagnosed as lung metastasis of cervical squamous cell carcinoma after evaluation by doctors. Six chemotherapy including paclitaxel, cisplatin and bevacizumab (Ankoda) were given. Continuous follow-up included clinical evaluation and SCC examination, as well as chest, abdominal and pelvic CT reexamination every 3 months.

25 months after the operation, a nodule with a diameter of 1.5 cm ∗ 1 cm was found above the left labia. PET-CT revealed a left inguinal lymph node of 1.0 cm ∗ 0.9 cm, and SCC value up to 2.2 ng/mL. HPV 16 and HPV 58 were still positive, and squamous cell carcinoma of the cervix with vulvar metastasis was considered.

**Fig. 1:**
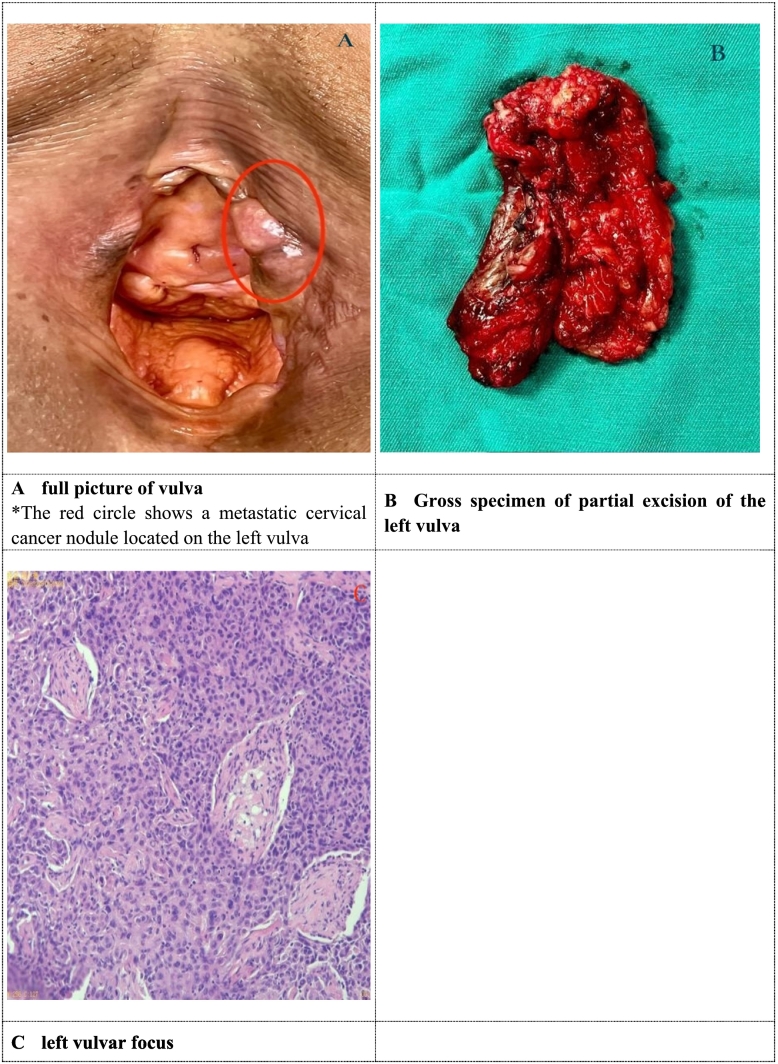
Vulvar gross view and pathology of vulvar tubercles. A Full picture of vulva. *The red circle shows a metastatic cervical cancer nodule located on the left vulva. B Gross specimen of partial excision of the left vulva. C Left vulvar focus. (For interpretation of the references to colour in this figure legend, the reader is referred to the web version of this article.)

The patient was then treated again in our hospital, and most of the vulva, bilateral inguinal lymph nodes, and posterior vaginal tubercles were removed. During the operation, a diameter of 1.5 cm ∗ 1 cm above the left labia and a diameter of 1 cm*1 cm on the posterior vaginal wall were visible. Intraoperative freeze pathological examination and postoperative pathology showed that the poorly differentiated squamous cell carcinoma invaded the left perineal clitoris with the depth about 5 mm. The tubercles of the posterior vaginal wall are chronic inflammation of the mucous membranes (Fig. 1).

The patients underwent 28 cycles of radiation therapy, 5 cycles of paclitaxel and cisplatin chemotherapy, 2 cycles of immunotherapy, and 2 cycles of traditional Chinese medicine therapy. After 12 radiotherapy sessions, the left leg developed swelling from the ankle to the knee and still completed radiotherapy. Follow-up included clinical assessment and SCC examinations, pelvic and abdominal MRI examinations every 3 months. At the time of submission, 19 months after surgery, the patient remained stable, except for persistent left leg swelling.

## 3 Discussion

We searched the Medline and Embase, PubMed databases for articles published from 2000 to 2024, using the keywords “cervical cancer”, “skin metastasis” and “vulva”. A total of 9 patients with vulvar metastasis of cervical cancer were found. See Table 1 including our patient, there are 10 cases of cervical cancer vulvar metastasis. The pathological characteristics, disease stage, disease progress, treatment and survival outcome of these patients were analyzed (Table 1).

The average age of the patients was 44.7 years (35–60 years). According to FIGO staging system, there were 5 cases (50 %) in I B stage, 2 cases (20 %) in II A stage and 2 cases (20 %) in III B stage. One case (10 %) was in IV stage. The most common pathological type was squamous cell carcinoma in 7 cases (70 %), followed by adenocarcinoma in 3 cases (30 %). Skin nodules were the first symptom in 5 cases (50 %), followed by plaques in 4 cases (40 %) and ulceration and edema1 in 10 %. One woman developed plaques and vesicaes at the same time (10 %) One woman had plaques and erosions (10 %), one woman had plaques, ulceration and edema (10 %) and one woman had plaques, Perkeratosis and ulceration (10 %). Radiotherapy was performed in 1 case (10 %) and radiotherapy and chemotherapy in 6 cases (60 %) after operation. No operation was performed, only radiotherapy and chemotherapy was performed in 2 cases (20 %), and only palliative treatment plus radiotherapy was performed in 1 case (10 %) because of the late detection period.

In the past 9 cases, 2 cases (20 %) were lost, the average survival time was 9 months (4–13 months). Our case currently survived for 13 months. The patients were generally in good condition. At present, there were only positive symptoms of swelling of the left leg. SCC was normal and was still under follow-up. The average time from initial diagnosis to skin metastasis was 20.7 months (0–78 months).

The average interval calculated by stages was 14.2 months in stage I, 60 months in stage II, 6 months in stage III and 4 months in stage IV. The longest reporting time of skin metastases is 6.5 years and the shortest is 0.That is to say, there is skin disease at the time of diagnosis. At the time of writing, nine women had died of illness (90 %) and one woman (10 %) was still ill.

From the review of the above literature, we found that vulvar masses were more common in cases of cervical cancer with vulvar metastasis. The interval between early cervical cancer and vulvar metastasis was longer than that of advanced cervical cancer. Multiple therapy and individualized treatment may prolong the survival time of patients.

Previous studies believed that the metastasis of cervical cancer was mainly direct diffusion and lymph node metastasis, and blood metastasis was rare. Another mechanism had been suggested in the literature, that was, vulvar skin metastasis was associated with radiotherapy. Possible causes include radiation-induced endothelial cell damage and the capture of tumor cells to form “tumor-induced vesicles.” It may be that the tumor changes the lymphatic pathway, blocks the lymphatic pathway, and shunts the lymphatic pathway to the skin [15].

In our case, tests were negative for HPV at first, and when the cancer had spread to the vulva, the test results were positive for HPV 16 and HPV 58, indicating the progression of HPV infection. False negative HPV results could occur for a variety of reasons, including test methods and viral load issues. Sampling errors, contamination of samples with blood or lubricants, and storage for too long can also lead to false negative HPV [16].

## 4 Conclusion

As a common malignant tumor of the female reproductive system, cervical cancer demonstrates diverse metastatic patterns that significantly impact prognosis and treatment strategies. Although vulvar metastasis is rare, its occurrence often indicates advanced disease and requires careful clinical evaluation. The low incidence of vulvar metastasis may lead to insufficient clinical awareness and vigilance. Additionally, patients may feel reluctant to report vulvar symptoms due to embarrassment or a lack of recognition of their significance, contributing to diagnostic delays and potential underreporting of such cases in clinical practice. To mitigate these challenges, clinicians should prioritize careful examination of the vulvar region when assessing the condition to avoid missing important clinical information. Meanwhile, treatment plans must consider both primary and metastatic foci, necessitating individualized strategies that may include adjustments to radiotherapy fields and the integration of multimodal therapies, such as surgery and chemotherapy. Therefore, an optimal therapeutic outcome could be obtained consequently.

## Author contribution

Ze-lan Liao: data collection, writing the paper.

Ka-na Wang: financial support, revising the paper.

Jia-wen Zhang: research design, data collection, data analysis and interpretation.

## Consent

Written informed consent was obtained from the patient for publication of this case report and accompanying images. A copy of the written consent is available for review by the Editor-in-Chief of this journal on request.

## Ethical approval

Strict adherence to the Declaration of Helsinki and the international ethical norms for scientific research were strictly adhered to. Approved by the Ethics Committee of West China Second Hospital of Sichuan University Ethics Approval No.: Medical Research 2019 Ethics Approval No. (Shen 23).

## Guarantor

Jia-wen Zhang.

## Research registration number

Not applicable.

## Funding

This study was supported by the Natural Science Foundation of Sichuan Province (No. 2022NSFSC0790).

## Conflict of interest statement

All authors declare that there is no conflict of interest.
